# A Novel Transfer Support Matrix Machine for Motor Imagery-Based Brain Computer Interface

**DOI:** 10.3389/fnins.2020.606949

**Published:** 2020-11-23

**Authors:** Yan Chen, Wenlong Hang, Shuang Liang, Xuejun Liu, Guanglin Li, Qiong Wang, Jing Qin, Kup-Sze Choi

**Affiliations:** ^1^School of Computer Science and Technology, Nanjing Tech University, Nanjing, China; ^2^Key Laboratory of Child Development and Learning Science, Ministry of Education, Southeast University, Nanjing, China; ^3^Smart Health Big Data Analysis and Location Services Engineering Lab of Jiangsu Province, Nanjing University of Posts and Telecommunications, Nanjing, China; ^4^CAS Key Laboratory of Human-Machine Intelligence-Synergy Systems, Shenzhen Institutes of Advanced Technology, Chinese Academy of Sciences, Shenzhen, China; ^5^Guangdong-Hong Kong-Macao Joint Laboratory of Human-Machine Intelligence-Synergy Systems, Shenzhen Institutes of Advanced Technology, Chinese Academy of Sciences, Shenzhen, China; ^6^School of Nursing, The Hong Kong Polytechnic University, Hong Kong, China

**Keywords:** motor imagery, brain-computer interface, electroencephalography, support matrix machine, transfer learning

## Abstract

In recent years, emerging matrix learning methods have shown promising performance in motor imagery (MI)-based brain-computer interfaces (BCIs). Nonetheless, the electroencephalography (EEG) pattern variations among different subjects necessitates collecting a large amount of labeled individual data for model training, which prolongs the calibration session. From the perspective of transfer learning, the model knowledge inherent in reference subjects incorporating few target EEG data have the potential to solve the above issue. Thus, a novel knowledge-leverage-based support matrix machine (KL-SMM) was developed to improve the classification performance when only a few labeled EEG data in the target domain (target subject) were available. The proposed KL-SMM possesses the powerful capability of a matrix learning machine, which allows it to directly learn the structural information from matrix-form EEG data. In addition, the KL-SMM can not only fully leverage few labeled EEG data from the target domain during the learning procedure but can also leverage the existing model knowledge from the source domain (source subject). Therefore, the KL-SMM can enhance the generalization performance of the target classifier while guaranteeing privacy protection to a certain extent. Finally, the objective function of the KL-SMM can be easily optimized using the alternating direction method of multipliers method. Extensive experiments were conducted to evaluate the effectiveness of the KL-SMM on publicly available MI-based EEG datasets. Experimental results demonstrated that the KL-SMM outperformed the comparable methods when the EEG data were insufficient.

## Introduction

Brain-computer interface (BCI) systems enable machines to accurately perceive the mental states of human beings, thereby establishing an effective user interface between humans and machines. There are several kinds of BCI paradigms, such as steady-state visual evoked potentials (Allison et al., 2008), P300 (Salvaris and Sepulveda, 2009), and motor imagery (MI) (Pfurtscheller and Neuper, 2001). Among them, the MI-based BCI is widely used because of its self-paced fashion, and it does not require any external stimuli (Pfurtscheller and Da Silva, 1999). Electroencephalography (EEG) is the most extensively used technique to record neuronal activity in the brain due to its high temporal resolution, portability, and non-invasiveness. EEG-based motor imagery BCI has shown great potential in many applications, such as rehabilitating the sensory-motor functions of disabled patients (Ang et al., 2011; Al-Qaysi et al., 2018) and facilitating smart living for healthy people (Vourvopoulos et al., 2017; Wang et al., 2019).

Although many machine learning algorithms have been developed to implement MI-based BCI with great success, most of them need to collect a considerable amount of labeled EEG data for model training, which is exceedingly time-consuming and labor-intensive. Insufficient labeled EEG data weaken the generalization capability of the classifier in the prediction. An intuitive solution to this problem is to leverage historical EEG data from the source domain (source subject) in modeling the target domain (target subject). However, this approach may engender some challenges. Owing to the EEG pattern variations between different subjects (Morioka et al., 2015), directly using the EEG data of the source domain may cause performance degradation. Furthermore, because the original EEG data contains personal information, the data of other subjects may not always be available for constructing the classifier for privacy reasons (Agarwal et al., 2019). Thus, exploring an effective knowledge transfer strategy that can protect the personal information of a source subject is highly desirable in the MI-based BCI.

From the perspective of transfer learning (Pan and Yang, 2009), the model knowledge of the source domain can potentially be leveraged to address these problems. Generally, EEG-based learning methods involve two steps: EEG feature extraction and classification. The model knowledge of the source domain can either be integrated into the feature extraction process (Kang et al., 2009; Samek et al., 2013), or be used in modeling the classifier (Azab et al., 2019). Specifically, Kang et al. (2009) proposed leveraging the linear combination of covariance matrices of the source subjects as reference during the feature extraction of the target EEG data. Azab et al. (2019) proposed the construction of multiple-source models and transfer of the weighted multiple-source model knowledge to the target domain. Deng et al. (2013) proposed a knowledge-leverage-based fuzzy system that can leverage the model knowledge from the source domain in order to make up for the lack of labeled target data as well as privacy protection.

Although it has been empirically demonstrated that the aforementioned methods are effective in dealing with EEG classification in scenarios where the labeled data are limited, these methods always need to transform the input data into vectors before classification. It is well known that EEG signals record brain activities over a period of time from multiple channels, which are naturally represented as matrices. Transforming the input matrices into vectors may destroy the correlation of rows or columns within matrix-form EEG features. Thus, several classification methods that can directly handle these matrix-form data have been developed accordingly. For example, Wolf et al. (2007) proposed modeling the regression matrix of a support vector machine (SVM), which is the sum of the *k* rank-one orthogonal matrices (rank-*k* SVM). Pirsiavash et al. (2009) proposed a bilinear SVM (BSVM) based on factorizing the regression matrix into the product of two low-rank matrices. Although these methods can capture the correlation within matrix data, pre-determining the rank of the regression matrix requires a tedious tuning procedure. Luo et al. (2015) proposed combining the nuclear norm and squared Frobenius norm of the regression matrix to derive the support matrix machine (SMM). The cornerstone of the SMM uses the nuclear norm of the regression matrix as the convex approximation of the matrix rank; thus, its optimization problem becomes more tractable and can be solved using the alternating direction method of multipliers (ADMM) method. Based on SMM, Zheng et al. proposed multiclass SMM (Zheng et al., 2018c) and sparse SMM (Zheng et al., 2018b) for EEG data. Although existing matrix classification methods can effectively deal with the matrix-form EEG data, they have not taken the transferrable knowledge into consideration to improve EEG classification performance. They may suffer from the weak generalization capability when the available EEG data are insufficient.

We propose a novel knowledge-leverage-based matrix classification method for MI-based EEG classification at the first time. The proposed knowledge-leverage-based SMM (KL-SMM) can address the above-mentioned problems by integrating the model knowledge from the source domain and a few labeled target EEG data. It possesses the powerful capability of the SMM for learning matrix-form data. Furthermore, the model knowledge of the source domain can be used to compensate for the deficiency in learning due to the lack of labeled target EEG data. Different from most current model parameter transfer learning methods, the proposed method can propagate the structural information from the source model to the target model. Hence, the generalization capability can be greatly enhanced by transferring the model knowledge and structural information of the source domain. Instead of directly using the source EEG data, the KL-SMM can afford privacy protection by leveraging only the model knowledge of the source domain. In addition, it can be efficiently optimized through the ADMM method. We conducted extensive experiments on two publicly available EEG datasets to validate the effectiveness of the proposed method. As demonstrated by the experimental results, the KL-SMM can achieve promising results in scenarios with few labeled target EEG data.

The remainder of this paper is organized as follows: Section “Related Works” is a review of related works. In Section “Matrix Learning Preliminaries”, the notations and preliminaries of the SMM are introduced. The KL-SMM model and its learning algorithm are described in Section “Knowledge-Leverage-Based SMM”. In Section “Experiments”, the details of extensive experiments and analyses are presented. The conclusions of the paper are presented in Section “Conclusion”.

## Related Works

Transfer learning has emerged as a novel technique for retaining and reusing knowledge learned from historical tasks for new tasks. As described above, transfer learning generally refers to the knowledge-leverage-based learning mechanism, which can extract useful knowledge from the source domain and propagate them as the supervision information for modeling the target domain. According to the types of transferred knowledge of the source domain, most current research on transfer learning for EEG classification can be broadly divided into the following categories: (1) instance transfer, (2) feature representation transfer, and (3) model parameter transfer (Wang et al., 2015).

For the first category, it is assumed that the partial source EEG data can be selected and considered together with few labeled target EEG data. The source EEG data are obtained through either instance selection or importance sampling cross-validation (Li et al., 2010; Hossain et al., 2016, 2018; Zanini et al., 2018). For example, Hossain et al. (2016) proposed an instance selection strategy based on active learning. The selected source EEG data were then used together with available target-labeled EEG data to train the target model. Li et al. (2010) demonstrated the possibility of weighing the source EEG data through the importance sampling cross-validation strategy, following which the source data with high weights were used to estimate the target classifier.

The aim of the feature representation transfer method is to learn a good feature representation, which has some relevant source knowledge encoded within it, for the target subject. Most feature representation transfer learning methods were developed based on the common spatial patterns (CSP) through the modification of the covariance matrix or optimization function (Kang et al., 2009; Lotte and Guan, 2010; Samek et al., 2013). For example, Samek et al. (2013) developed an extension of the CSP. They proposed learning a stationary subspace in which the stationary information of multiple subjects can be transferred. In addition to the above-mentioned shallow feature representation transfer learning methods, several deep transfer learning methods (Fahimi et al., 2019; Hang et al., 2019) have been proposed. In general, these methods apply the domain adaptation techniques in a task-specific layer to incorporate the learned source and target deep features into a common feature space. For example, Hang et al. (2019) proposed leveraging the maximum mean discrepancy and the center-based discriminative feature learning techniques simultaneously to reduce the domain shift, demonstrating a performance improvement in the MI-based BCI.

The third category is the model parameter transfer, which assumes that the source subjects and target subjects share some parameters or prior distributions of the models. Model parameter transfer learning methods always leverage source models to model the target subjects in EEG classification. For example, Azab et al. (2019) proposed a logistic regression-based transfer learning method. The linear combination of multiple-source models was transferred for the construction of the target domain. In Alamgir et al. (2010); Jayaram et al. (2016), Alamgir et al. proposed a multi-task learning method to improve the generalization performance of the EEG classification for individual subjects using subject-specific information, as well as the shared model knowledge inherent in all available subjects.

Despite these successes, most current transfer learning methods require the direct use of source EEG data, which may cause the issue of privacy disclosure, especially for biomedical information. Furthermore, existing transfer learning methods used for EEG recognition always built on that the input data are vectors. However, transforming EEG data, which are naturally represented as matrices, into vectors will destroy their structural information. The proposed method belongs to the third category. Unlike the previous transfer learning methods, the KL-SMM can incorporate model knowledge from the source domain, thereby guaranteeing privacy protection to some extent; it can also directly handle matrix-form EEG data.

## Matrix Learning Preliminaries

Among the current matrix learning methods, the SMM (Luo et al., 2015) and its variants [e.g., (Zheng et al., 2018a)] are applied in many fields, owing to their simplicity and effectiveness. In this section, we present some notations and preliminary knowledge on the SMM, which are the foundation of the proposed KL-SMM method.

### Mathematical Notations

Matrices are denoted by bold uppercase letters (i.e., **X**) in the following. For a matrix **X** ∈ *ℝ*^*d*_1_×*d*_2_^ of rank *r*, it can be expressed as *rank*(**X**) = *r*. The condensed singular value decomposition (SVD) of **X** is denoted as $\text{X}={\text{U}}_{X}\,\sum_{X}{\text{V}}_{X}^{T}$, where **U**_*X*_ ∈ *ℝ*^*d*_1_×*r*^ and **V**_*X*_ ∈ *ℝ*^*d*_2_×*r*^ satisfy ${\text{U}}_{X}^{T}\,{\text{U}}_{X}={\text{I}}_{r}$ and ${\text{V}}_{X}^{T}\,{\text{V}}_{X}={\text{I}}_{r}$, and ∑_**X**_ = diag(σ_1_(**X**),σ_2_(**X**),…,σ_*r*_(**X**)) with σ_1_(**X**)≥σ_2_(**X**)≥⋯≥σ_*r*_(**X**) > 0.

**Definition 1.** Given any τ > 0, the singular value thresholding (SVT) (Cai et al., 2010) of matrix **X** is defined as

(1) $$D_{\tau }\,\left[X\right]={\text{U}}_{X}\,{\text{S}}_{\tau }\,\left[\sum_{X}\right]{\text{V}}_{X}^{T}$$

where *S*_τ_[∑_**X**_] = diag({σ_1_(**X**)−τ} +,{σ_2_(**X**)−τ} +,…,{σ_*r*_(**X**)−τ} +) and {*z*} + = *max*⁡(*z*,0).

Let ${∥X∥}_{F}=\sqrt{\sum_{i=1}^{r}\sigma_{i}\,{\left(X\right)}^{2}}$ be the Frobenius norm of **X**, ${∥X∥}_{*}=\sum_{i=1}^{r}\sigma_{i}\,\left(X\right)$ denotes the nuclear norm of **X**, and the subdifferential of ∥**X**∥_*_ can be defined as follows.

**Definition 2.** The subdifferential of ∥**X**∥_*_, that is, ∂⁡∥**X**∥_*_ (Candes and Recht, 2009), can be formulated as

$$\partial ∥X{∥}_{*}=\{{\text{U}}_{X}{\text{V}}_{X}^{T}+\text{Z}|\text{Z}\in {\mathbb{R}}^{d_{1}\times d_{2}},{\text{U}}_{X}^{T}\text{Z}=0,$$

(2) $$\;\;{\text{ZV}}_{X}=0,∥Z{∥}_{2}\leq 1\},$$

where ∥**Z**∥_2_ = σ_1_(**Z**) denotes the spectral norm of **Z**.

### SMM

The matrix classifier, SMM, is defined as a penalty function plus a hinge loss. The penalty function, i.e., spectral elastic net, which enjoys the property of grouping effect as well as keeping a low-rank representation. The hinge loss enjoys the property of large margin while contributing to the sparseness and robustness of the classifier. The objective function of the SMM can be formulated as follows:

(3) $$\begin{array}{cc} \min_{w,b} & \frac{1}{2}\,t\,r\,\left({\text{W}}^{T}\,\text{W}\right)+\tau \,{∥W∥}_{*}+C\,\sum_{i=1}^{N}\xi_{i}\\ s.t. & y_{i}\,\left[t\,r\,\left({\text{W}}^{T}\,{\text{X}}_{i}\right)+b\right]\geq 1-\xi_{i},\xi_{i}\geq 0,\forall i=1,2,\ldots ,N \end{array}.$$

Specifically, the spectral elastic net can be represented as a combination of the squared Frobenius matrix norm ${∥W∥}_{F}^{2}=t\,r\,\left({\text{W}}^{T}\,\text{W}\right)$ and nuclear norm ∥**W**∥_*_ on the regression matrix *W*.

The objective function of the SMM can be solved through the ADMM method; thus, Eq. (3) is reformulated as

(4) $$\begin{array}{c} \min_{W,b,S}F\,\left(W,b\right)+G\,\left(S\right)\\ s.t.\text{S}-\text{W}=0 \end{array},$$

where $F\left(W,b\right)=\frac{1}{2}tr\left({\text{W}}^{T}\text{W}\right)+C\sum_{i=1}^{N}\{1-y_{i}[tr\left({\text{W}}^{T}{\text{X}}_{i}\right)$ + *b*]} + and *G*(**S**) = τ∥**S**∥_*_.

According to the augmented Lagrangian function in Eq. (4), (**W**,*b*) and **S** can be iteratively computed in two steps:

(5) $$\left({\text{W}}^{\left(k\right)},b^{\left(k\right)}\right)=\underset{\left(W,b\right)}{\text{argmin}}F\,\left(W,b\right)-t\,r\,\left({\left(\Lambda^{\left(k-1\right)}\right)}^{T}\,\text{W}\right)\;+\frac{\rho }{2}\,{∥\text{W}-{\text{S}}^{\left(k-1\right)}∥}_{F}^{2},$$

(6) $${\text{S}}^{\left(k\right)}=\underset{S}{\arg\min}G\,\left(S\right)+t\,r\,\left({\left(\Lambda^{\left(k-1\right)}\right)}^{T}\,\text{S}\right)+\frac{\rho }{2}\,{∥{\text{W}}^{\left(k\right)}-\text{S}∥}_{F}^{2},$$

where *k* denotes the iteration index. ρ > 0 is a hyperparameter, and **Λ** is a Lagrangian multiplier.

### Knowledge-Leverage-Based SMM

Generally, the current SMM and its variants belong to the data-driven method that always focuses on achieving impressive classification performance with sufficient training data. In practice, it is necessary to collect sufficient EEG data for each subject to establish a subject-specific classifier. However, long-term recording EEG data may exhaust the subject. Therefore, to model the target domain using insufficient EEG data, we proposed a novel algorithm to enhance the generalization capability of the SMM on the target domain by leveraging the useful knowledge underlying the source domain.

**FIGURE 1:**
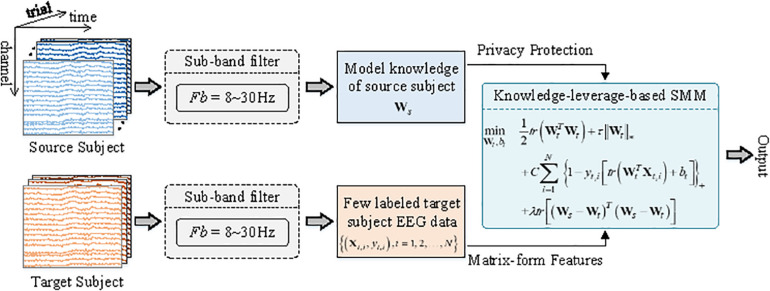
Framework of the proposed KL-SMM for EEG-based motor imagery BCI. Both source EEG data and target EEG data are first bandpass filtered in the frequency range of 8∼30 Hz. SMM is then applied to learn source model ***W*_*s*_**. The source model (only needed) enables the classification model realize the privacy protection. An objective function of the proposed KL-SMM is then implemented by using the source model and the matrix-form features extracted from few labeled target data.

The framework of the KL-SMM for an EEG-based MI BCI is illustrated in Figure 1. To model the target domain, two main types of information, the model knowledge of the source domain and few labeled target EEG data, are used simultaneously.

#### KL-SMM Model

A dataset *D*_*s*_ = {(**X**_*s*,1_,*y*_*s*,1_),(**X**_*s*,2_,*y*_*s*,2_),⋯,(**X**_*s*,*N*_*s*__,*y*_*s*,*N*_*s*__)} in source domain, it consists of *N*_*s*_ trials labeled EEG signals. **X**_*s*,*i*_ ∈ *ℝ*^*p*×*q*^ denotes the *i*th trial with *p*×*q* dimension. *y*_*s,i*_ is the corresponding class label of **X**_*s*,*i*_. A dataset *D*_*t*_ = {(**X**_*t*,1_,*y*_*t*,1_),⋯,(**X**_*t*,*N*_,*y*_*t*,*N*_),**X**_*t*,*N* + 1_,⋯,**X**_*t*,*N*_*t*__} in the target domain, it consists of *N* labeled EEG trials and (*N*_*t*_−*N*) unlabeled trials, where *N*≪(*N*_*t*_−*N*).

For modeling the target domain, we proposed to integrate the labeled target EEG data and source model as follows:

(7) $$\min_{f}\sum_{i=1}^{N}L\,\left(f\,\left({\text{X}}_{t,i}\right),y_{t,i}\right)+\lambda \,t\,r\,\left[{\left({\text{W}}_{s}-{\text{W}}_{t}\right)}^{T}\,\left({\text{W}}_{s}-{\text{W}}_{t}\right)\right],$$

Here, *L*(⋅,⋅) denotes the loss function. $f\,\left({\text{X}}_{t,i}\right)=t\,r\,\left({\text{W}}_{t}^{T}\,{\text{X}}_{t,i}\right)+b$ denotes the matrix classifier to be learned. Eq. (7) includes two terms, where the first term is used to learn from labeled target EEG data, and the second term is designed to leverage the model knowledge (i.e., **W**_*s*_) underlying the source domain. The goal is to exploit the desired KL-SMM by approximating its model to the source model. The parameter λ is adopted to balance the influence between the two terms above.

As in the SMM, we introduced the spectral elastic net penalty to capture the correlation information within the matrix-form EEG data. Furthermore, the hinge loss function was adopted, owing to its inherent characteristic of sparseness and robustness. Above all, the objective function of the proposed KL-SMM can be formulated as follows:

(8) $$\begin{array}{cc} \min_{W_{t},b_{t}} & \frac{1}{2}\,t\,r\,\left({\text{W}}_{t}^{T}\,{\text{W}}_{t}\right)+\tau \,{∥{\text{W}}_{t}∥}_{*}+C\,\sum_{i=1}^{N}\xi_{i}^{t}\\ & +\lambda \,t\,r\,\left[{\left({\text{W}}_{s}-{\text{W}}_{t}\right)}^{T}\,\left({\text{W}}_{s}-{\text{W}}_{t}\right)\right]\\ s.t. & y_{t,i}\,\left[t\,r\,\left({\text{W}}_{t}^{T}\,{\text{X}}_{t,i}\right)+b_{t}\right]\geq 1-\xi_{i}^{t},\xi_{i}^{t}\geq 0,\\ & \forall i=1,2,\ldots ,N \end{array},$$

where the parameter *C* > 0 is used to maintain a balance between fitting the labeled target EEG data and minimizing the complexity of the solution.

#### Parameter Learning for KL-SMM

Because the Eq. (8) is convex in both **W**_*t*_ and *b*_*t*_, an alternating iterative strategy based on the ADMM method can be used to derive the learning algorithm of the KL-SMM. Specifically, by introducing an auxiliary variable *S*_*t*_, the objective function of the KL-SMM can be equivalently reformulated as

(9) $$\begin{array}{cc} \min_{W_{t},b_{t},S_{t}} & F\,\left({\text{W}}_{t},b_{t}\right)+G\,\left({\text{S}}_{t}\right)\\ s.t. & {\text{S}}_{t}-{\text{W}}_{t}=0 \end{array},$$

where $F\left({\text{W}}_{t},b_{t}\right)=\frac{1}{2}tr\left({\text{W}}_{t}^{T}{\text{W}}_{t}\right)+C\sum_{i=1}^{N}\{1-y_{t,i}[tr\left({\text{W}}_{t}^{T}{\text{X}}_{t,i}\right)$ + *b*_*t*_]}+ + λ*tr*[(**W**_*s*_−**W**_*t*_)*T*(**W**_*s*_−**W**_*t*_)] and *G*(**S**_*t*_) = τ∥**S**_*t*_∥_*_.

The parameter optimization of Eq. (9) can be solved using the augmented Lagrangian algorithm

$$L_{a}\,\left({\text{W}}_{t},b_{t},{\text{S}}_{t},\Lambda \right)=F\,\left({\text{W}}_{t},b_{t}\right)+G\,\left({\text{S}}_{t}\right)+t\,r\,\left[\Lambda^{T}\,\left({\text{S}}_{t}-{\text{W}}_{t}\right)\right]$$

(10) $$+\frac{\rho }{2}\,{∥{\text{S}}_{t}-{\text{W}}_{t}∥}_{F}^{2},$$

where **Λ** is the Lagrangian multiplier, and ρ > 0 is a hyperparameter. Theorems 1 and 2 provide the calculations of parameters **S**_*t*_ and (**W**_*t*_,*b*_*t*_).

**Theorem 1.** For the fixed **W**_*t*_, using the Lagrangian multiplier **Λ** and any positive scalar τ > 0, ρ > 0 in Eq. (10), **S**_*t*_ can be optimized using the following update rule:

(11) $${\text{S}}_{t}=D_{\tau }\,\left[\rho \,{\text{W}}_{t}-\Lambda \right].$$

*Proof of Theorem 1*: Supposing **W**_*t*_ is fixed, the optimization problem in Eq. (10) is equivalent to minimizing the function as follows:

(12) $$J\,\left({\text{S}}_{t}\right)=\tau \,{∥{\text{S}}_{t}∥}_{*}+t\,r\,\left(\Lambda^{T}\,{\text{S}}_{t}\right)+\frac{\rho }{2}\,{∥{\text{S}}_{t}-{\text{W}}_{t}∥}_{F}^{2}.$$

Because *J*(**S**_*t*_) is convex, with respect to **S**_*t*_, if $0\in \partial J\,\left({\text{S}}_{t}^{*}\right)$ with ${\text{S}}_{t}^{*}=\left(1\,/\,\rho \right)\cdot D_{\tau }\,\left[\rho \,{\text{W}}_{t}-\Lambda \right]$ can be proven, we can conclude that ${\text{S}}_{t}^{*}$ is a solution to Eq. (12). The derivation of *J*(**S**_*t*_) with respect to **S**_*t*_ can be expressed as

(13) $$\partial J\,\left({\text{S}}_{t}\right)=\Lambda -\rho \,{\text{W}}_{t}+\rho \,{\text{S}}_{t}+\tau \cdot \partial {∥{\text{S}}_{t}∥}_{*}.$$

To further simplify this equation, let the SVD of (ρ**W**_*t*_−**Λ**) be denoted as $\rho \,{\text{W}}_{t}-\Lambda ={\text{U}}_{a}\,\sum_{a}{\text{V}}_{a}^{T}+{\text{U}}_{b}\,\sum_{b}{\text{V}}_{b}^{T}$. In the equation, ∑_*a*_ represents the diagonal matrix with diagonal entries greater than τ. ∑_*b*_ represents the remaining part of the SVD with diagonal entries less than or equal to τ. **U**_*a*_ and **V**_*a*_ (**U**_*b*_ and **V**_*b*_) are matrices that correspond to the left and right singular vectors of the diagonal matrix ∑_*a*_ (∑_*b*_). In terms of Definition 1, ${\text{S}}_{t}^{*}$ can be reformulated as $\left(1\,/\,\rho \right)\cdot {\text{U}}_{a}\,\left(\sum_{a}-\tau \,\text{I}\right)\,{\text{V}}_{a}^{T}$. Substituting ${\text{S}}_{t}^{*}$ into Eq. (13), we have

(14) $$\begin{array}{cc} {\partial J\,\left({\text{S}}_{t}\right)|}_{S_{t}^{*}} & =\Lambda -\rho \,{\text{W}}_{t}+\rho \,{\text{S}}_{t}^{*}+\tau \cdot \partial {∥{\text{S}}_{t}^{*}∥}_{*}\\ & =-\tau \,\left({\text{U}}_{a}\,{\text{V}}_{a}^{T}+\frac{1}{\tau }\,{\text{U}}_{b}\,\sum_{b}{\text{V}}_{b}^{T}\right)+\tau \cdot \partial {∥{\text{S}}_{t}^{*}∥}_{*} \end{array}.$$

Let $\text{Z}=\left(1\,/\,\tau \right)\cdot {\text{U}}_{b}\,\sum_{b}{\text{V}}_{b}^{T}$, because **U**_*a*_, **U**_*b*_, **V**_*a*_, **V**_*b*_ are column orthogonal, we can easily verify that ${\text{U}}_{a}^{T}\,\text{Z}=0$, **ZV**_*a*_ = 0, and ∥**Z**∥_2_≤1. Thus, we have $0\in \partial J\,\left({\text{S}}_{t}^{*}\right)$. Theorem 1 is proved.

**Theorem 2.** For the fixed **S**_*t*_, (**W**_*t*_,*b*_*t*_) can be optimized using the following update rule:

(15) $${\text{W}}_{t}=\frac{1}{2\,\lambda +\rho +1}\,\left(\Lambda +\rho \,{\text{S}}_{t}+2\,\lambda \,{\text{W}}_{s}+\sum_{i=1}^{N_{t}}\alpha_{i}\,y_{t,i}\,{\text{X}}_{t,i}\right),$$

(16) $$b_{t}=\frac{1}{\left|\Delta \right|}\,\sum_{i\in \Delta }\left\{y_{t,i}-\left[t\,r\,\left({\text{W}}_{t}^{T}\,{\text{X}}_{t,i}\right)\right]\right\},$$

where Δ = {*i*|0≤α_*i*_≤*C*,∀*i* = 1,2,…,*N*} refers to the Lagrangian multipliers, andα = [α_1_,α_2_,⋯,α_*N*_]*T* ∈ *ℝ*^*N*^ can be obtained using the box constraint quadratic programming solver:

(17) $$\begin{array}{cc} \max_{\alpha } & -\frac{1}{2}\,\alpha^{T}\,\text{K}\,\alpha +{\text{H}}^{T}\,\alpha \\ s.t\,.0 & \leq \alpha \leq C,\alpha^{T}\,\text{Y}=0 \end{array},$$

where **K** = [*K*_*ij*_] ∈ *ℝ*^*N*×*N*^ and **H** = [h_*i*_] ∈ *ℝ*^*N*^ with

(18) $$K_{i\,j}=\frac{1}{2\,\lambda +\rho +1}\,y_{t,i}\,y_{t,j}\,t\,r\,\left({\text{X}}_{t,i}^{T}\,{\text{X}}_{t,j}\right),$$

(19) $${\text{h}}_{i}=1-\frac{1}{2\,\lambda +\rho +1}\,y_{t,i}\,t\,r\,\left[{\left(\Lambda +\rho \,{\text{S}}_{t}+2\,\lambda \,{\text{W}}_{s}\right)}^{T}\,{\text{X}}_{t,i}\right].$$

*Proof of Theorem 2*: Given the fixed variable **S**_*t*_, the optimization problem in Eq. (10) equals to optimize the following objective function:

(20) $$\begin{array}{cc} \min_{W_{t},b_{t}} & \frac{1}{2}\,t\,r\,\left({\text{W}}_{t}^{T}\,{\text{W}}_{t}\right)+C\,\sum_{i=1}^{N}\xi_{i}^{t}-t\,r\,\left(\Lambda^{T}\,{\text{W}}_{t}\right)+\frac{\rho }{2}\,{∥{\text{S}}_{t}-{\text{W}}_{t}∥}_{F}^{2}\\ & +\lambda \,t\,r\,\left[{\left({\text{W}}_{s}-{\text{W}}_{t}\right)}^{T}\,\left({\text{W}}_{s}-{\text{W}}_{t}\right)\right]\\ s.t. & y_{t,i}\,\left[t\,r\,\left({\text{W}}_{t}^{T}\,{\text{X}}_{t,i}\right)+b_{t}\right]\geq 1-\xi_{i}^{t},\xi_{i}^{t}\geq 0,\forall i=1,2,\ldots ,N \end{array}.$$

The augmented Lagrangian function of Eq. (20) is denoted as

(21) $$\begin{array}{cc} L_{a}^{\prime } & =\frac{1}{2}\,t\,r\,\left({\text{W}}_{t}^{T}\,{\text{W}}_{t}\right)+C\,\sum_{i=1}^{N}\xi_{i}^{t}-t\,r\,\left(\Lambda^{T}\,{\text{W}}_{t}\right)+\frac{\rho }{2}\,{∥{\text{S}}_{t}-{\text{W}}_{t}∥}_{F}^{2}\\ & +\lambda \,t\,r\,\left[{\left({\text{W}}_{s}-{\text{W}}_{t}\right)}^{T}\,\left({\text{W}}_{s}-{\text{W}}_{t}\right)\right]\\ & -\sum_{i=1}^{N}\alpha_{i}\,\left\{y_{t,i}\,\left[t\,r\,\left({\text{W}}_{t}^{T}\,{\text{X}}_{t,i}\right)+b_{t}\right]-1+\xi_{i}^{t}\right\}-\sum_{i=1}^{N}\beta_{i}\,\xi_{i}^{t} \end{array}.$$

Setting the derivative of $L_{a}^{\prime }$ with respect to $\xi_{i}^{t}$ and *b*_*t*_, to 0, we can obtain

(22) $$\frac{\partial L_{a}^{\prime }}{\partial \xi_{i}^{t}}=0\Rightarrow C-\alpha_{i}-\beta_{i}=0\,\text{and}\,\frac{\partial L_{a}^{\prime }}{\partial b_{t}}=0\Rightarrow \sum_{i=1}^{N}\alpha_{i}\,y_{t,i}=0.$$

Substituting Eq. (22) into Eq. (21), and then setting the derivative of $L_{a}^{\prime }$ with respect to *W*_*t*_ to 0, we obtain

$$\frac{\partial L_{a}^{\prime }}{\partial {\text{W}}_{t}}=0\Rightarrow {\text{W}}_{t}$$

(23) $$=\frac{1}{2\,\lambda +\rho +1}\,\left(\Lambda +\rho \,{\text{S}}_{t}+2\,\lambda \,{\text{W}}_{s}+\sum_{i=1}^{N}\alpha_{i}\,y_{t,i}\,{\text{X}}_{t,i}\right).$$

Substituting Eq. (22) and Eq. (23) into Eq. (21),

$$L_{a}^{\prime }=\sum_{i=1}^{N}\left(1-\frac{1}{2\,\lambda +\rho +1}\,y_{t,i}\,t\,r\,\left[{\left(\Lambda +\rho \,{\text{S}}_{t}+2\,\lambda \,{\text{W}}_{s}\right)}^{T}\,{\text{X}}_{i}\right]\right)\,\alpha_{i}$$

(24) $$-\frac{1}{2\,\left(2\,\lambda +\rho +1\right)}\,\sum_{i=1}^{N}\sum_{j=1}^{N}\alpha_{i}\,\alpha_{j}\,y_{t,i}\,y_{t,j}\,t\,r\,\left({\text{X}}_{t,i}^{T}\,{\text{X}}_{t,j}\right)+\theta .$$

Here, θis a constant, which can be represented as $\theta =\frac{\rho }{2}\,t\,r\,\left({\text{S}}_{t}^{T}\,{\text{S}}_{t}\right)+\lambda \,t\,r\,\left({\text{W}}_{s}^{T}\,{\text{W}}_{s}\right)-\frac{1}{2\,\left(2\,\lambda +\rho +1\right)}\,{∥\Lambda +\rho \,{\text{S}}_{t}+2\,\lambda \,{\text{W}}_{s}∥}_{F}^{2}$. Thus, the dual problem of Eq. (24) can be denoted as

(25) $$\begin{array}{cc} \max_{\alpha } & \sum_{i=1}^{N}\left(1-\frac{1}{2\,\lambda +\rho +1}\,y_{t,i}\,t\,r\,\left[{\left(\Lambda +\rho \,{\text{S}}_{t}+2\,\lambda \,{\text{W}}_{s}\right)}^{T}\,{\text{X}}_{i}\right]\right)\,\alpha_{i}\\ & -\frac{1}{2\,\left(2\,\lambda +\rho +1\right)}\,\sum_{i=1}^{N}\sum_{j=1}^{N}\alpha_{i}\,\alpha_{j}\,y_{t,i}\,y_{t,j}\,t\,r\,\left({\text{X}}_{t,i}^{T}\,{\text{X}}_{t,j}\right)\\ s.t. & \sum_{i=1}^{N}\alpha_{i}\,y_{t,i}=0,0\leq \alpha_{i}\leq C,\forall i=1,2,\ldots ,N \end{array}.$$

**Algorithm 1:** The learning procedure for KL-SMM

**Input:** Training dataset $D_{T}={\left\{{\text{X}}_{t,i},y_{t,i}\right\}}_{i=1}^{N}$, source model **W**_*s*_, parameter τ and λ;

**Output:** **W**_*t*_, *b*_*t*_;

**Initialize:** ${\text{S}}_{t}^{\left(-1\right)}={\hat{\text{S}}}_{t}^{\left(0\right)}\in {\text{R}}^{p\times q}$, $\Lambda^{\left(-1\right)}={\hat{\Lambda }}^{\left(0\right)}\in {\text{R}}^{p\times q}$, *v*^(1)^ = 1, η ∈ (0,1), ρ > 0, *l=1*


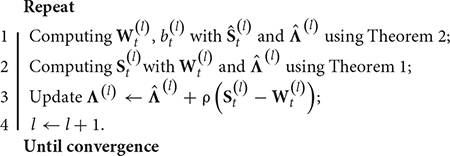


The optimization problem of Eq. (21) can finally be transformed into a QP problem. Substituting the obtained optimal solution α into Eq. (23), it is easy to obtain the value of **W**_*t*_. Finally, the optimal *b*_*t*_ can be calculated as follows:

(26) $$b_{t}=y_{t,i}-\left[t\,r\,\left({\text{W}}_{t}^{T}\,{\text{X}}_{t,i}\right)\right].$$

In practice, averaging these optimal solutions, we can obtain

(27) $$b_{t}=\frac{1}{\left|\Delta \right|}\,\sum_{i\in \Delta }\left\{y_{t,i}-\left[\text{tr}\,\left({\left({\text{W}}_{t}\right)}^{T}\,{\text{X}}_{t,i}\right)\right]\right\},$$

where Δ = {*i*|0≤α_*i*_≤*C*,∀*i* = 1,2,…,*N*}. Theorem 2 is proved.

For the fixed **W**_*t*_ and **S**_*t*_, the Lagrangian multiplier **Λ** in Eq. (10) can be updated as follows:

(28) $$\Lambda^{\left(k\right)}=\Lambda^{\left(k-1\right)}+\rho \,\left({\text{S}}_{t}-{\text{W}}_{t}\right).$$

The optimal solution is estimated iteratively. The learning procedure for the KL-SMM is given in Algorithm 1.

#### Computational Complexity

We further analyzed the computational complexity of the KL-SMM. In Algorithm 1, Step 1 computes the parameter (**W**_*t*_,*b*_*t*_) by solving a QP problem in Eq. (17), which takes time *O*(*N*^2^*pq*) with *N*samples of *p*×*q* dimension. Step 2 computes the eigen decomposition for **S**_*t*_ in Eq. (11), which takes time *O*(*min*⁡(*p*^2^*q*,*pq*^2^)). In practice, the dimensions, *p* and *q*, of the extracted EEG features are not too high. Thus, the computational complexity of the KL-SMM is dominated by the QP, that is, *O*(*I*⋅*N*^2^*pq*), where *I* denotes the iteration number.

## Experiments

In this section, we evaluate the proposed KL-SMM on two publicly available MI EEG datasets [i.e., Datasets IIa and IIb of the BCI competition (Hang et al., 2020)], which can be found in http://www.bbci.de/competition/iv/. We first describe the EEG datasets. Then, the compared methods and corresponding parameter settings are provided. Finally, we present and discuss the experimental results.

### EEG Data Description and Preprocessing

(1) *BCI competition IV Dataset IIa (Exp.1)*: This dataset includes 22-channel EEG signals recorded from nine subjects (denoted as *S01–S09*). During the experiment, each subject was required to perform four kinds of MI tasks, hand (left and right), foot, and tongue. A total of 576 trials of two sessions on different days were collected for each subject. In our experiment, we used the left-hand and right-hand EEG data. In addition, the EEG trials collected from the second day were adopted. Thus, the training and test datasets each contained 72 EEG trials.
(2) *BCI competition IV Dataset IIb (Exp.2)*: This dataset also contains the EEG signals of nine subjects (denoted as *B01–B09*), which were recorded using three electrodes, *C3*, *Cz*, and *C4*. During the experiment, each subject was instructed to perform left- and right-hand MI tasks for 4.5 s. For each subject, there were five sessions. Sessions 1, 2, and 3 were collected on the first day, and 4 and 5 were collected on the second day. Similar to *Exp.1*, the EEG trials collected from the second day were used. Specifically, Session 4 was used as the training data, and Session 5 was used as the test data.

With reference to Hang et al. (2020), there was a time interval of *[0.5, 3]*s after the visual cues in each trial for all the datasets. We bandpass-filtered the EEG signals to 8–30 Hz through a five-order Butterworth filter, which covers the dominated frequency band for MI tasks (Nam et al., 2011). Then, we adopted the spatial filters to detect the MI-related desynchronization/synchronization (ERD/ERS) patterns. Finally, the widely used band-power estimation method (Vidaurre et al., 2005) was used to extract the matrix-form EEG features for all the subjects. To construct the transfer learning tasks, each subject was considered the target domain, and the training data of the remaining subjects constituted the source domain. To evaluate the performance of the KL-SMM, we set three different numbers of labeled target EEG data, that is, the first 8, 14, and 20 training trials. The classification performances on the test data of all the subjects were reported.

### Experimental Setup

#### Baseline Methods

To evaluate the classification performance of the aforementioned transfer tasks, the proposed method was compared with four methods in the experiments: (1) SVM (Vapnik, 1995), (2) BSVM (Pirsiavash et al., 2009), (3) SMM (Luo et al., 2015), and (4) Adaptive SVM (ASVM) (Yang et al., 2007).

#### Implementation Details

It is known that the format of the input data of both the SVM and ASVM should be vectors or scalars. Thus, we first had to reshape the extracted two-dimensional matrix features into vector features. For the BSVM, SMM, and proposed KL-SMM, the matrix features can be inputted directly. To evaluate the effect of the transfer learning mechanism, because the SVM, BSVM, and SMM are no-transfer baselines, we simply used the labeled target EEG trials as the training data to build these classification models. In addition, for the ASVM and KL-SMM, we also leveraged the source model knowledge in constructing the target classifier. However, unlike the ASVM, the traditional transfer learning method, the KL-SMM can directly process EEG matrix features and fully exploit the structural information.

The optimal parameters were selected using a five-fold cross-validation method on the training group for all comparison methods. Parameter *C* was assigned by selecting the value from the set {1*e*−6, *1e-5*, *1e-4*, *1e-3*, 1*e*−2, 1*e*−1, 1*e*0, 1*e*1}. For the SMM and KL-SMM, we tuned the parameter τ from the set {1*e*−5, *2e-5*, *5e-5*, *1e-4*, *2e-4*, *5e-4*, *1e-3*, *2e-3*, *5e-3*, 1*e*−2, *2e-2*, *5e-2*, 1*e*−1, *2e-1*, *5e-1*, 1*e*0}. To ensure a fair comparison with the ASVM, an adjustable parameter λ_*A*_ was added to the term of knowledge transfer, which can control how much the knowledge of source domain to transfer. For the KL-SMM and ASVM, parameters λ and λ_*A*_ were set by selecting the value from set {1*e*−4, 5*e*−4, 1*e*−3, 5*e*−3, *1e-2*, *5e-2*, *1e-1*, *5e-1*, 1*e*0}. To validate the classification performance of our method, we used the following metrics on the test dataset, i.e., Accuracy (ACC), *F1* score (F1), and the area under the receiver operating characteristics curve (AUC). Herein, *ACC* = (*TP* + *TN*)/(*TP* + *FN* + *FP* + *TN*) and *F1* = 2×PPV×SEN/(*PPV* + *SEN*), where the positive predictive value (PPV) = TP/(*TP* + *FP*) and sensitivity (SEN) = TP/(*TP* + *FN*).

### Experimental Results Analysis

The classification performances of all the comparison methods on 14 labeled target EEG trials on two datasets are given in Tables 1–6. The performance comparison of the KL-SMM with the compared methods in *Exp.1* is shown in Tables 1–3. The classification results of all compared methods in *Exp. 2* are shown in Tables 4–6. The best classification performance values are highlighted in bold. According to the results, the following conclusions can be drawn.

**TABLE 1:** Comparison of ACC using 14 labeled target EEG data in *Exp.1*.

	Target subjects									
Methods	S01	S02	S03	S04	S05	S06	S07	S08	S09	Avg.
SVM	0.5972	0.5000	0.9583	0.5556	0.5139	0.5556	0.5972	0.9306	0.8472	0.6728
BSVM	0.5556	0.4861	0.9583	0.5278	0.5972	0.5417	0.5972	**1.0000**	0.8472	0.6790
SMM	0.6111	0.5000	**0.9722**	0.5556	0.5556	0.5556	0.6111	0.9444	0.8472	0.6836
ASVM	0.6111	**0.5139**	0.9167	0.6667	0.6389	0.5972	0.7222	0.9444	0.8472	0.7176
KL-SMM	**0.6528**	0.5000	0.9306	**0.7361**	**0.6944**	**0.6111**	**0.7361**	0.9583	**0.8611**	**0.7423**

**TABLE 2:** Comparison of AUC using 14 labeled target EEG data in *Exp.1*.

	Target subjects									
Methods	S01	S02	S03	S04	S05	S06	S07	S08	S09	Avg.
SVM	0.6265	0.5015	0.9776	0.5640	0.5316	0.5548	0.6019	0.9228	0.9190	0.6889
BSVM	0.5980	0.5023	0.9367	0.5239	0.6019	0.5579	0.6181	**1.0000**	0.9190	0.6953
SMM	0.6273	0.5015	**0.9830**	0.5640	0.5733	0.5548	0.6142	0.9406	0.9190	0.6975
ASVM	0.6273	**0.5177**	0.9313	0.6790	0.6481	0.6142	0.7261	0.9406	0.9190	0.7337
KL-SMM	**0.6744**	0.5015	0.9375	**0.7261**	**0.7068**	**0.6173**	**0.7415**	0.9606	**0.9213**	**0.7541**

**TABLE 3:** Comparison of F1 using 14 labeled target EEG data in *Exp. 1*.

	Target subjects									
Methods	S01	S02	S03	S04	S05	S06	S07	S08	S09	Avg.
SVM	0.3256	0.5385	0.9589	0.4483	0.3636	0.4667	0.6234	0.9275	0.8308	0.6092
BSVM	0.2000	0.5195	0.9565	0.3462	0.5538	0.4407	0.5397	**1.0000**	0.8308	0.5986
SMM	0.3636	0.5385	**0.9722**	0.4483	0.4286	0.4667	0.6410	0.9429	0.8308	0.6258
ASVM	0.3636	**0.5570**	0.9091	0.6842	0.6579	**0.5672**	0.6970	0.9429	0.8308	0.6900
KL-SMM	**0.4681**	0.5385	0.9254	**0.7397**	**0.7027**	0.5333	**0.7164**	0.9565	**0.8485**	**0.7143**

**TABLE 4:** Comparison of ACC using 14 labeled target EEG data in *Exp. 2*.

	Target subjects									
Methods	B01	B02	B03	B04	B05	B06	B07	B08	B09	Avg.
SVM	0.4750	0.5125	0.4500	0.8688	0.5813	0.6688	0.7000	0.9500	0.7250	0.6590
BSVM	0.5125	0.5125	0.4813	0.8750	0.5688	0.5813	0.6250	0.9375	0.7188	0.6458
SMM	0.4875	**0.5313**	0.4500	0.8750	0.5938	0.6750	0.6813	0.9563	0.7625	0.6681
ASVM	0.6063	0.5000	**0.5000**	0.9125	0.6063	0.6938	0.6813	0.9500	0.6688	0.6799
KL-SMM	**0.6125**	**0.5313**	0.4625	**0.9563**	**0.6438**	**0.7125**	**0.7250**	**0.9625**	**0.7375**	**0.7049**

**TABLE 5:** Comparison of AUC using 14 labeled target EEG data in *Exp. 2*.

	Target subjects									
Methods	B01	B02	B03	B04	B05	B06	B07	B08	B09	Avg.
SVM	0.4614	0.5247	0.4655	0.8698	0.5948	0.6761	0.6980	0.9577	0.7263	0.6638
BSVM	0.4963	0.5102	0.5048	0.8652	0.5781	0.5833	0.6159	0.9453	0.7080	0.6452
SMM	0.4747	0.5431	0.4655	0.8814	0.6081	0.6825	0.6841	0.9605	**0.7678**	0.6742
ASVM	0.5922	0.5103	**0.5356**	0.9377	0.6172	0.7138	0.6664	0.9577	0.6580	0.6876
KL-SMM	**0.5939**	**0.5470**	0.4697	**0.9673**	**0.6627**	**0.7389**	**0.7202**	**0.9670**	0.7416	**0.7120**

**TABLE 6:** Comparison of F1 using 14 labeled target EEG data in *Exp. 2*.

	Target subjects									
Methods	B01	B02	B03	B04	B05	B06	B07	B08	B09	Avg.
SVM	0.5172	0.4348	0.3125	0.8800	0.6082	0.6788	0.7303	0.9518	0.7755	0.6544
BSVM	0.6100	**0.5938**	0.3566	0.8837	0.5767	0.5839	0.7170	0.9390	0.7368	0.6664
SMM	0.5119	0.4604	0.3125	0.8851	0.6061	0.6829	0.7052	0.9576	**0.7935**	0.6572
ASVM	0.6358	0.3651	**0.5918**	0.9176	0.6557	0.7322	0.7243	0.9518	0.7039	0.6976
KL-SMM	**0.6702**	0.4000	0.4691	**0.9571**	**0.6885**	**0.7527**	**0.7528**	**0.9634**	0.7857	**0.7155**

From the classification performances of all the comparison methods on the 14 labeled target EEG trials, we found that the proposed KL-SMM method achieved the highest average results in terms of the ACC, AUC, and F1. As shown in Tables 1–3, the proposed KL-SMM outperformed the baseline SMM on average by 5.87%, 5.66%, and 8.85% based on the ACC, AUC, and F1, respectively. As can be observed from the classification results in Tables 4–6, the KL-SMM outperformed the SMM on average by 3.68%, 3.78%, and 5.83% based on the ACC, AUC, and F1, respectively. The promising performances prove that the KL-SMM can leverage the model knowledge of the source subject to boost the generalization capability of the SMM when there are limited labeled EEG trials. In addition, the KL-SMM boosted the classification accuracy for six out of nine subjects in Exp.1, and eight out of nine subjects in Exp.2, respectively. These experimental results further demonstrate the effectiveness of the proposed KL-SMM that leveraged the knowledge underlying the source domain.

The BSVM and SMM outperformed the SVM in most cases. This confirms the ability of the BSVM and SMM to exploit the correlations between rows or columns of EEG matrix features. Notably, the SMM has better classification performance than the BSVM because of its convex objective function that can be effectively optimized by the ADMM method. Furthermore, it can be observed that, owing to its ability to leverage the source model knowledge in modeling the target domain when the labeled target EEG data is very limited, the ASVM yielded better classification results than the SVM. The foundation of the KL-SMM is the SMM, which can leverage the source model knowledge and exploit the structural information within the EEG feature matrices. The experimental results prove that structural information can indeed improve classification performance.

**FIGURE 2:**
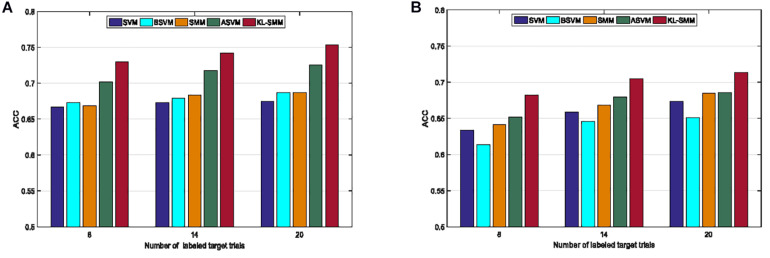
Average classification ACCs of comparison methods with different numbers of labeled target EEG trials in *Exp.1* **(A)** and *Exp.2* **(B)**.

We further studied the effects of different numbers of labeled target EEG instances on the classification performance of the KL-SMM. Figure 2 shows the average classification ACCs when 8, 14, and 20 labeled target EEG trials were available from the target subject. Figures 2A,B show the average classification results of all the compared methods for *Exp.1* and *Exp.2*, respectively. It can be observed that the KL-SMM outperformed the other methods in all the cases. Specifically, the improvement was more pronounced when few labeled target EEG trials were available, as shown in Figure 2B. From these results, we can observe that increasing the number of the labeled target EEG instances improved the average classification ACCs of all the compared methods. This is mainly because more training data may enhance the generalization performance of the classification model. The average ACC of the KL-SMM was significantly better than those of the other methods when there was no knowledge transfer, especially when the labeled target EEG instances were very limited. Overall, compared to other methods, the classification performance of the proposed KL-SMM was superior. The encouraging results were mainly attributed to the fact that the KL-SMM method possessed the matrix learning capability derived from the matrix learning machine, which allowed it to directly handle the matrix-form features, thus retaining the structural information of EEG data. In addition, the KL-SMM achieved a more outstanding classification performance because of its ability to leverage the useful model knowledge of the source domain.

## Discussion

### Statistical Analysis

We further performed a *t*-test statistical analysis to verify whether there was a significant difference with a confidence level of 95% between the KL-SMM and the other methods. The results of the *t*-test using different numbers of labeled target EEG trials are shown in Table 3. A *p*-value less than 0.05 indicates that there are significant differences between the KL-SMM and the other methods. We highlighted the statistically significant differences in boldface. From Table 7, it can be observed that in all cases, the null hypothesis can be rejected. This proves that the KL-SMM significantly outperformed the other methods. This further demonstrated the ability of the KL-SMM to capture the structural information within the EEG data, in addition to a strong transfer learning capability. Therefore, it is suitable for the classification of complex matrix-form EEG data with cross-subject variability.

**TABLE 7:** Statistical significance comparisons of ACC of KL-SMM and other methods in *Exp.1* and *Exp.2*.

	Exp.1				Exp.2			
Num. of labeled trials	KL-SMM vs. SVM	KL-SMM vs. BSVM	KL-SMM vs. SMM	KL-SMM vs. ASVM	KL-SMM vs. SVM	KL-SMM vs. BSVM	KL-SMM vs. SMM	KL-SMM vs. ASVM
8	**0.0167**	**0.0116**	**0.0085**	**0.0085**	**0.0164**	**0.0107**	**0.0465**	**0.0307**
14	**0.0287**	**0.0485**	**0.0442**	**0.0207**	**0.0129**	**0.0074**	**0.0419**	**0.0376**
20	**0.0039**	**0.0227**	**0.0060**	**0.0249**	**0.0014**	**0.0053**	**0.0215**	**0.0328**

### Running Time

**FIGURE 3:**
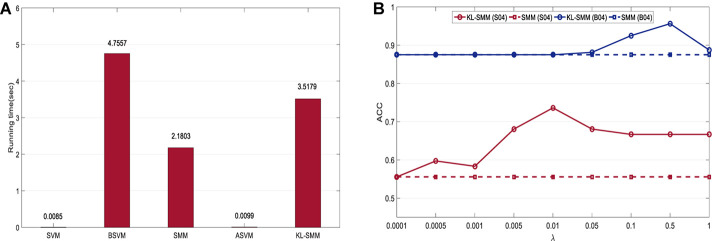
**(A)** Running time for subject *S01* using 14 labeled target EEG trials in *Exp.1*; **(B)** Parameter sensitivity of KL-SMM for the transfer tasks *S04* in *Exp.1* and *B04* in *Exp.2* using 14 labeled target EEG trials, respectively.

Figure 3A shows the running time of the KL-SMM and other methods on a subject, *S01*, in *Exp.1* using 14 labeled target EEG trials. Except for the SVM and ASVM, the KL-SMM achieved comparable computational cost with the traditional matrix leaning method SMM. Furthermore, the KL-SMM required less computational time, compared to the BSVM. It was proven that the running time of the KL-SMM was approximately 1.6 times less that of the SMM. The KL-SMM achieved better classification results, without the increase in computational costs becoming unacceptable. This shows the potential value of the KL-SMM for real-world BCI applications.

### Parameter Sensitivity

We further show the effect of free parameter on the performance of KL-SMM, i.e., the knowledge transfer penalty λ. We conduct parameter sensitivity experiments on the transfer tasks *S04* in *Exp.1* and *B04* in *Exp.2* using 14 labeled target EEG trials, respectively. We vary the parameter of interest in {1*e*−4, 5*e*−4, 1*e*−3, 5*e*−3, *1e-2*, *5e-2*, *1e-1*, *5e-1*, 1*e*0}. Figure 3B shows the classification accuracy of our KL-SMM in contrast to SMM represented as dashed lines. It can be found that the accuracy of KL-SMM is improved with the increase of parameter λ, suggesting that taking the model knowledge of source domain into account can benefit for EEG classification. As the parameter value is further increased, the classification performance will decrease due to the distribution discrepancy between the source domain and the target domain.

## Conclusion

In this study, we proposed a KL-SMM method for MI-based BCIs. The proposed KL-SMM belongs to the matrix classifier, which can exploit the structural information of EEG data in matrix form. Furthermore, it can leverage the existing source model knowledge in modeling the construction of the target subjects in scenarios of limited labeled target training data. Similar to the SMM, the KL-SMM can be easily optimized using the ADMM. Extensive experimental results on two publicly available MI datasets demonstrate the superiority of the KL-SMM to the compared methods in most cases. However, despite its promising performance, there is still room for improvement. For example, adaptively controlling the penalty λ is critical to determining how much knowledge is transferred. In addition, how to extend KL-SMM to multi-class classification will be investigated in future work.

## Data Availability Statement

Publicly available datasets were analyzed in this study. This data can be found here: http://www.bbci.de/competition/iv/.

## Author Contributions

YC is responsible for data processing and data analysis. WH and SL are responsible for manuscript writing. XL and QW is responsible for study design. GL is responsible for experimental design. JQ and K-SC are responsible for manuscript editing. All authors contributed to the article and approved the submitted version.

## Conflict of Interest

The authors declare that the research was conducted in the absence of any commercial or financial relationships that could be construed as a potential conflict of interest.
